# Adjuvant Docetaxel and Cyclophosphamide (DC) with Prophylactic Granulocyte Colony-Stimulating Factor (G-CSF) on Days 8 &12 in Breast Cancer Patients: A Retrospective Analysis

**DOI:** 10.1371/journal.pone.0107273

**Published:** 2014-10-15

**Authors:** Rinat Yerushalmi, Hadar Goldvaser, Aaron Sulkes, Irit Ben-Aharon, Daniel Hendler, Victoria Neiman, Noa Beatrice Ciuraru, Luisa Bonilla, Limor Amit, Alona Zer, Tal Granot, Shulamith Rizel, Salomon M. Stemmer

**Affiliations:** 1 Institute of Oncology, Davidoff Center, Rabin Medical Center, Beilinson Hospital, Petah-Tikva, Israel; 2 Sackler Faculty of Medicine, Tel-Aviv University, Tel-Aviv, Israel; Istituto Superiore di Sanità, Italy

## Abstract

**Purpose:**

Four cycles of docetaxel/cyclophosphamide (DC) resulted in superior survival than doxorubicin/cyclophosphamide in the treatment of early breast cancer. The original study reported a 5% incidence of febrile neutropenia (FN) recommending prophylactic antibiotics with no granulocyte colony-stimulating factor (G-CSF) support. The worldwide adoption of this protocol yielded several reports on substantially higher rates of FN events. We explored the use of growth factor (GF) support on days 8 and 12 of the cycle with the original DC protocol.

**Methods:**

Our study included all consecutive patients with stages I–II breast cancer who were treated with the DC protocol at the Institute of Oncology, Davidoff Center (Rabin Medical Center, Petah Tikva, Israel) from April, 2007 to March, 2012. Patient, tumor characteristics, and toxicity were reported. *Results*: In total, 123 patients received the DC regimen. Median age was 60 years, (range, 25–81 years). Thirty-three patients (26.8%) were aged 65 years and older. Most of the women (87%) adhered to the planned G-CSF protocol (days 8 &12). 96% of the patients completed the 4 planned cycles of chemotherapy. Six patients (5%) had dose reductions, 6 (5%) had treatment delays due to non-medical reasons. Thirteen patients (10.6%) experienced at least one event of FN (3 patients had 2 events), all requiring hospitalization. Eight patients (6.5%) required additional support with G-CSF after the first chemotherapy cycle, 7 because of FN and one due to neutropenia and diarrhea.

**In Conclusion:**

Primary prophylactic G-CSF support on days 8 and 12 of the cycle provides a tolerable option to deliver the DC protocol. Our results are in line with other retrospective protocols using longer schedules of GF support.

## Introduction

Adjuvant chemotherapy for patients with early-stage breast cancer has been shown to improve both disease-free survival and overall survival [1]. However, selecting the most suitable chemotherapy regimen for each patient remains a challenge. Many of the adjuvant protocols in use are based on anthracyclines and hence harbor potential cardiotoxicity. Jones and colleagues studied an optional non-anthracycline adjuvant regimen. They found that 4 cycles of docetaxel/cyclophosphamide (DC) produced a superior survival rate compared with doxorubicin/cyclophosphamide (AC) in the treatment of early-stage breast cancer. The study reported that 5% of the patients developed fever and neutropenia events using prophylactic antibiotics with no granulocyte colony-stimulating factor (G-CSF) support [2]. The worldwide adoption of this protocol yielded several reports on significantly higher rates of febrile neutropenia (FN) events (up to 80% in a subgroup of patients in that report) [3], prompting the addition G-CSF by most medical centers [3]–[6].

The schedule of G-CSF administration varies among oncologists' practice. Previous studies have suggested that switching from pegfilgrastim to just a few doses of filgrastim might be enough to adequately support adjuvant chemotherapy. Compared with daily filgrastim administration for 7 days (days 8 to 14), fewer doses of 2 (days 8 and 12) and 4 (days 8, 10, 12, and 14) days showed less side effects such as bone pain and incidence of fever [7], [8]. We explored the use of only 2 doses of filgrastim (Neupogen) 5 µg/kg on days 8 &12 with the original DC protocol. This report summarizes treatment-related morbidity of the DC protocol with G-CSF support given on days 8 &12 on each cycle.

## Patients and Methods

### Study design

The study was approved by the institutional review board of Rabin Medical Center. Consent was not obtained (as was approved by the ethic committee). Patient information was anonymized and de-identified prior to analysis.

This retrospective analysis included all consecutive patients with stage I–II breast cancer who were treated with the DC protocol (docetaxel 75 mg/m^2^ and cyclophosphamide 600 mg/m^2^ intravenously on day 1 every 21 days for 4 cycles) and received filgrastim (Neupogen, Amgen Inc. Thousand Oaks, CA. United States) 5 µg/kg subcutaneously on days 8 &12 of each cycle (for at least one cycle) at the Institute of Oncology, Davidoff Center, Rabin Medical Center, Petah Tikva, Israel from April 2007 to March 2012. No prophylactic antibiotic treatment was added.

### Statistical analysis

Descriptive statistics were used to summarize patient and tumor characteristics and treatments received. Chi-squared test was used to compare the number of FN events between age groups (≤65, >65 years). *P*<0.05 was considered significant.

## Results

### Study population

Of the 131 patients who received the DC regimen in our institution within the study timeframe, 123 were included in the current analysis (8 were excluded: 5 did not receive G-CSF support, 2 received pegfilgrastim, 1 received 5 filgrastim injections). Median (range) age was 60 (25–81) years and 33 patients (26.8%) were >65 years of age (**Table 1**).

**Table 1:** Patient and tumor characteristics.

No. of patients	N = 123	% 100
**Age Median (range), years**	60 (25–81)	
**T stage**		
T1a	1	<1
T1b	21	17
T1c	64	52
T2	37	30
**N stage**		
N0	85	69
N 1 mic	7	5.7
N 1 (without N1 mic)	29	23.6
N2,3	1	<1
**Hormonal receptor status**		
Estrogen receptor positive	109	89
Progesterone receptor positive	37	30
* **Positive HER2 Status**	0	100
**Grade**		
I	6	4.9
II	65	53
III	52	42

*HER2 positive cases were defined as IHC+ 3 or if IHC+ 2, FISH with amplification ratio ≥2.0.

### Chemotherapy and G-CSF exposure

One hundred eighteen patients (95.9%) completed the 4 planned DC cycles (of whom 2 patients received 6 cycles according to their physician's initial treatment plan). Five patients (4.1%) discontinued treatment after the first DC cycle due to chest pain, second malignancy, allergy, diarrhea and abdominal pain, or relocation (1 patient each). Of 496 planned cycles, 15 (3.0%) were missed. Six patients (4.9%) required dose reduction for ≥1 cycle (9%–25% reduction of the planned dose), and 6 patients (4.9%) had a treatment delay of up to 5 days, none due to medical reasons.

The majority of patients (107 patients; 87.0%) adhered to the planned filgrastim protocol (days 8 &12). Fourteen patients (11.4%) required additional G-CSF support after the first DC cycle, 13 because of FN and 1 due to neutropenia and diarrhea. The median (range) age of the patients requiring additional G-CSF support was 58 (28–73) years. In 5 patients (4.1%), the filgrastim dose was decreased after the first DC cycle to only 1 injection per DC cycle due to leukocytosis and severe bone pain (**Table 2**).

**Table 2:** **G**-CSF use: Deviation from the planned D1,8 treatment program.

	Deviation	Number of patients (N = 16)	Age (years)
**Increasing G-CSF support following cycle 1**	3 cycles with 3 filgrastim injections and prophylactic antibiotics	1	58
	3 cycles with 4 filgrastim injections	4	46,63,65,73
	3 cycles with pegfilgrastim	3	28,42,50
**Increasing G-CSF support following cycle 3**	1 cycle with pegfilgrastim	2	51,54
**Increasing G-CSF support twice**	4 filgrastim injections after cycle two and pegfilgrastim after cycle three	1	60
**Reducing G-CSF support after cycle 1**	2 filgrastim injections in cycle 1 and reduction to 1 filgrastim injection in at least one of the cycles thereafter	5	47,59,61,61,67

*G-CSF* granulocyte colony-stimulating factor.

### Toxicity

Thirteen patients (10.6%) experienced at least one FN event (3 patients had 2 events), all requiring hospitalization. Eight patients (6.5%) experienced the FN event in their first DC cycle. In total, FN events occurred in 16 of 496 cycles (3.2%). The rates of FN events were similar in older (>65 years) and younger (≤65 years) patients (4 of 33 patients [12.1%] and 9 of 90 patients [10.0%], respectively; *P* = 0.74). Overall, the median (range) age of the patients requiring hospitalization due to FN was 60 (42–73) years. Median age of the older hospitalized group was 72 years compared to 54 years in the younger group. In addition to the 13 FN-related hospitalizations, 14 more hospitalizations were reported due to other reasons including: diarrhea, chest pain, fever, and cellulitis (2 patients each), as well as chronic obstructive pulmonary disease, tonsillitis, bone pain, hearing problem, observation after treatment, and an unknown reason (1 patient each). Of note, 4 additional patients experienced grade 2–3 diarrhea but did not require hospitalization. There was no treatment related mortality.

## Discussion

Adjuvant systemic chemotherapy has a critical impact on many patients' survival and their quality of life [1]. The combination of docetaxel and cyclophosphamide provides a reasonable option for patients with early-stage breast cancer, both estrogen receptor positive or negative and HER2-negative disease. For some patients, such as those who are not candidates for standard anthracycline-containing regimens, the DC regimen may be a preferred treatment [2]. Adherence to the original dose density/intensity of the chosen systemic treatment is highly important. FN can jeopardize the treatment dose and schedule and in extreme cases can be a life-threatening event. G-CSF support can reduce the risk of such life-threatening events [9]; therefore, the American Society of Clinical Oncology (ASCO), the National Comprehensive Cancer Network (NCCN), and the European Organization for Research and Treatment of Cancer (EORTC) endorse the use of G-CSF in certain situations [10]–[12]. The 2006 ASCO Update of Recommendations for the Use of White Blood Cell (WBC) Growth Factors and the NCCN 2011 guidelines state that primary prophylaxis with a white cell growth factor is recommended for the prevention of FN in patients with high risk of developing this complication of therapy. High risk is defined as a risk greater than 20%. The EORTC 2011 guidelines recommend G-CSF use when reductions in chemotherapy dose intensity or density may be detrimental to the patients' outcome as in the adjuvant setting.

Several studies assessed FN rates in breast cancer patients treated with the DC regimen (**Table 3**). The highest rates of FN events were found in a Japanese (28.3%) and in a Canadian (33%) studies [3], [4]. In both studies, there was no routine prophylactic G-CSF administration. The older group (age>65 years) suffered from an extremely high rate of FN events: eighty percent of the older patients in the Japanese study, in which no primary prophylaxis was offered, and 40% in the Canadian study, where only 28% of the patients had received prophylactic treatment. In the other studies, the total FN event rate was not more than 12% even in the older subgroup. In these studies, a large proportion (49%–100%) of the patients received primary G-CSF prophylaxis except in the Jones et al. study in which prophylactic antibiotics were recommended to all patients (though the authors declare that they do not know the precise number of patients who received antibiotic prophylactically) [2], [5], [6], [13]. Thus, our study is aligned with prior reports supporting the use of prophylactic G-CSF with the DC protocol, although the optimal G-CSF schedule is still to be determined.

**Table 3:** Patient characteristics and safety profile in various studies investigating the DC regimen in the breast cancer adjuvant setting.

	Jones et al. [2].	Chan et al [6]	The current study	Ngamphaiboon et al. [5]	Freyer et al. [13]	Takabatake et al [3]	Vandenberg et al. [4]
**Number of Patients**	506	159	123	111	110	53	39
**Median age**	50	49	60	56	73	54	65
**Range**	(27–64)	(25,71)	(25–81)	(27–79)	(70–85)	(33–67)	(39–84)
**Proportion of patients ≥65 years,%**	15.4	NA	26.8	22	100	9.4	21
**Comorbidities**	NA	CVD,34%	NA	CCI≥2, 19%	71% with ≥1 comorbidity	NA	31% with major comorbidity
**Primary G-CSF prophylaxis**	No	79.9% filgrastim 3 injections every other day	100% filgrastin, D8,12	100% Pegfilgrastim	49% Unknown type of G-CSF	No	28% filgrastim or pergfil-grastim
**Prophylactic antibiotics**	aYes	No	No	No	No	No	No
**Proportion of patients who received ≥4 cycles of DC**	93%	88%	96%	85.6%	91%	94.3%	92.3%
**Exposure to DC**	DI- 99.8%	Docetaxel	Dose reduction:	DI-91.2%	17% required dose & schedule modifications	Dose reduction 7.5%	NA
		mean dose:	4.9% of pts				
		73.4%.	Treatment delay:				
		Cyclophosphamide	4.9% of pts				
		Mean dose:					
		C-92.7%					
**Febrile Neutropenia, %**							
Overall	5	12.6	10.6	7	5^b^	28.3	33
<65 years	4	NA	10	4	NA	22.9	22.9
≥65 years	8	NA	12	8	NA	80	40

*CCI* Charlson comorbidity index, *CVD* Cardiovascular disease, *DC* docetaxel/cyclophosphamide, *DI* dose intensity, *G-CSF* granulocyte colony-stimulating factor, *NA* not available, *Pts* patients.

a Though it is unknown to the authors the precise number of patients who received antibiotic prophylactically.

b All patients were >70 years.

Whereas some oncologists recommend 5–7 daily filgrastim injections starting 48–72 hours after chemotherapy initiation, others recommend pegfilgrastim to achieve a long-acting support. Our patients received only 2 G-CSF injections as primary prevention treatment with comparable results to previous studies with different schedules of G-CSF support. Of note, Chan et al. provided a protocol similar to ours, a short primary prophylactic G-CSF protocol. There, the patients received 3 injections, every other day and had comparable results for FN events [6].

The benefit from G-CSF support seems to be substantial. A recent meta-analysis based on 61 randomized clinical trials with various tumor types and involving 24,796 patients which compared chemotherapy with and without G-CSF demonstrated that all-cause mortality with a median follow up of 3 years was significantly reduced with G-CSF support [14], further supporting its increasing use in clinical practice. However, G-CSF injections may have considerable side effects such as severe bone pain (which was the reason for hospitalization for one patient in the current study), allergic reaction, and fever [15].

In the last decade, several publications have reported an increase in the rate of G-CSF use in the adjuvant setting [16]–[19]. As expected, the adoption of a G-CSF support as a standard in the oncologic practice raises economic concerns [20]–[21]. Seven days of filgrastim treatment cost $500–800 and one injection of pegfilgrastim costs $1500–2000. As the DC protocol is based on 4 chemotherapy cycles, the financial burden associated with G-CSF support can reach $8000 per patient. Recently, the widespread use of the DC regimen with G-CSF support added to the budget constraints in the adjuvant treatment of patients with breast cancer. Younis et al. noted that the higher FN incidence associated with the DC regimen resulted, as expected, in less favorable cost-utility estimates. Adding G-CSF to all patients doubled the estimated cost per quality-adjusted life year (QALY) when comparing 4 cycles of DC to 4 cycles of AC [22]. It should be noted that FN events usually require hospitalization.

Our patients received only 2 G-CSF injections as primary prophylactic treatment with a lower rate of FN events as compared to some previous publications. However, 10.6% of the patients still experienced at least one FN event. This raises the question of whether more than 2 injections or upfront pegfilgrastim administration would further reduce the risk of such events.

The limitations of this study are its retrospective design and the use of a patient cohort from a single center. However, we included all consecutive patients who received the protocol and there was no missing data for any of the patients. Only 8 out of 131 patients (6%) were assigned to the DC regimen without the G-CSF studied protocol, of whom 5 patients did not get any G-CSF support. This precludes the potential bias that patients prone to develop FN were offered a different G-CSF protocol.

In summary, primary prophylactic growth factor support on days 8 & 12 provides a tolerable option to deliver the DC protocol. Our results are in line with other retrospective protocols using longer schedules of growth factor support. Further studies are required to determine the most appropriate G-CSF regimen for the DC protocol.
